# Revealing an Exceptional Case of Budd-Chiari Syndrome

**DOI:** 10.7759/cureus.69949

**Published:** 2024-09-22

**Authors:** Krishna Geetha Narne, Vaishnavi K.I.S.N, Jagadeswar Kakumani, Vivekanandan T, Gowri Shankar A

**Affiliations:** 1 Internal Medicine, Saveetha Medical College and Hospital, Saveetha Institute of Medical and Technical Sciences (SIMATS) Saveetha University, Chennai, IND

**Keywords:** budd-chiari syndrome, echinococcus granulosus, hepatic vein obstruction, hydatid cyst, inferior vena cava obstruction

## Abstract

Budd-Chiari syndrome (BCS) is a rare disorder characterized by hepatic venous outflow obstruction, leading to substantial effects, which include liver congestion, ascites, and liver failure. This is an unusual but very important form of secondary BCS caused by extraluminal compression from structures such as abscesses, tumors, or cysts. This case study exemplifies a 46-year-old female with no previous medical history who developed BCS due to the encasement of the IVC and hepatic veins secondary to a hydatid cyst, which is an uncommon presentation. Distension, ascites, and pain in the abdomen are the chief complaints. Investigations were positive for a hydatid cyst in the liver on imaging and serology, as well as features commensurate with BCS. The patient received conservative management with albendazole and anticoagulation. However, she refused cyst surgical resection and thus died three weeks after her discharge because of a cardiorespiratory arrest possibly associated with complications of her hydatid cyst like anaphylaxis secondary to rupture of the hydatid cyst. Therefore, this case brings out the significance of considering hydatid cysts when making a differential diagnosis on patients suffering from BCS, especially in endemic areas, and also underscores the importance of early recognition, treatment measures, and education to avoid fatal conditions.

## Introduction

Budd-Chiari syndrome (BCS) is an infrequent and grave condition characterized by the blockage of hepatic venous outflow, resulting in liver congestion, hepatomegaly, ascites, and potentially severe liver failure [1]. The exact prevalence of BCS is not known but is estimated to be 1/1,00,000 people in the general population, and it can be more in developing countries. It is classified into two types: primary and secondary. The primary leading cause of BCS is myeloproliferative disorders. Other causes include malignancy, hyper-coagulable states, drugs such as oral contraceptive pills, paroxysmal nocturnal hemoglobinuria, Behcet disease, etc. Parasitic liver diseases such as hydatid cysts or amebic liver abscesses and pyogenic liver abscesses are rare etiologic factors in secondary BCS. Thrombosis or webs within the hepatic veins are common causes of primary BCS, which often result from intraluminal obstructions. On the other hand, extraluminal compression or invasion by tumors, abscesses, or cysts is what leads to the blood flow of the liver being interfered with thus causing secondary BCS [2]. Hydatid disease, also known as echinococcosis, is an infection brought about by parasitic tapeworm Echinococcus species. The most prevalent pathogen that causes this condition is Echinococcus granulosus. This illness tends to be endemic in some areas where livestock keeping is practiced because hosts of parasites include dogs and livestock animals. Human beings become accidental intermediate hosts upon ingestion of parasite eggs developing hydatid cysts in different organs predominantly the liver and lungs. These cysts also increase over time and may cause symptoms if they compress adjacent structures or even burst [3]. Hydatid disease is a disease of the liver, and in 50-70% of cases, it manifests as hepatic cysts. Although most often asymptomatic, the size, location, secondary infections or rupture leads to significant complications [4]. In rare occasions such as the one described here compression of IVC due to hepatic hydatid cysts may obstruct liver blood flow leading to secondary BCS [5]. This case study presents an interesting but rare case of BCS resulting from a hydatid cyst located on the right lobe of the liver. This case prompts clinicians that hydatid disease should be included among BCS differentials, especially in Echinococcus endemic areas. The presentation of the patient, diagnostic workup, as well and management for this case demonstrate how difficult and essential the diagnosis and treatment are for these unusual complications related to echinococcus.

## Case presentation

**Figure 1:**
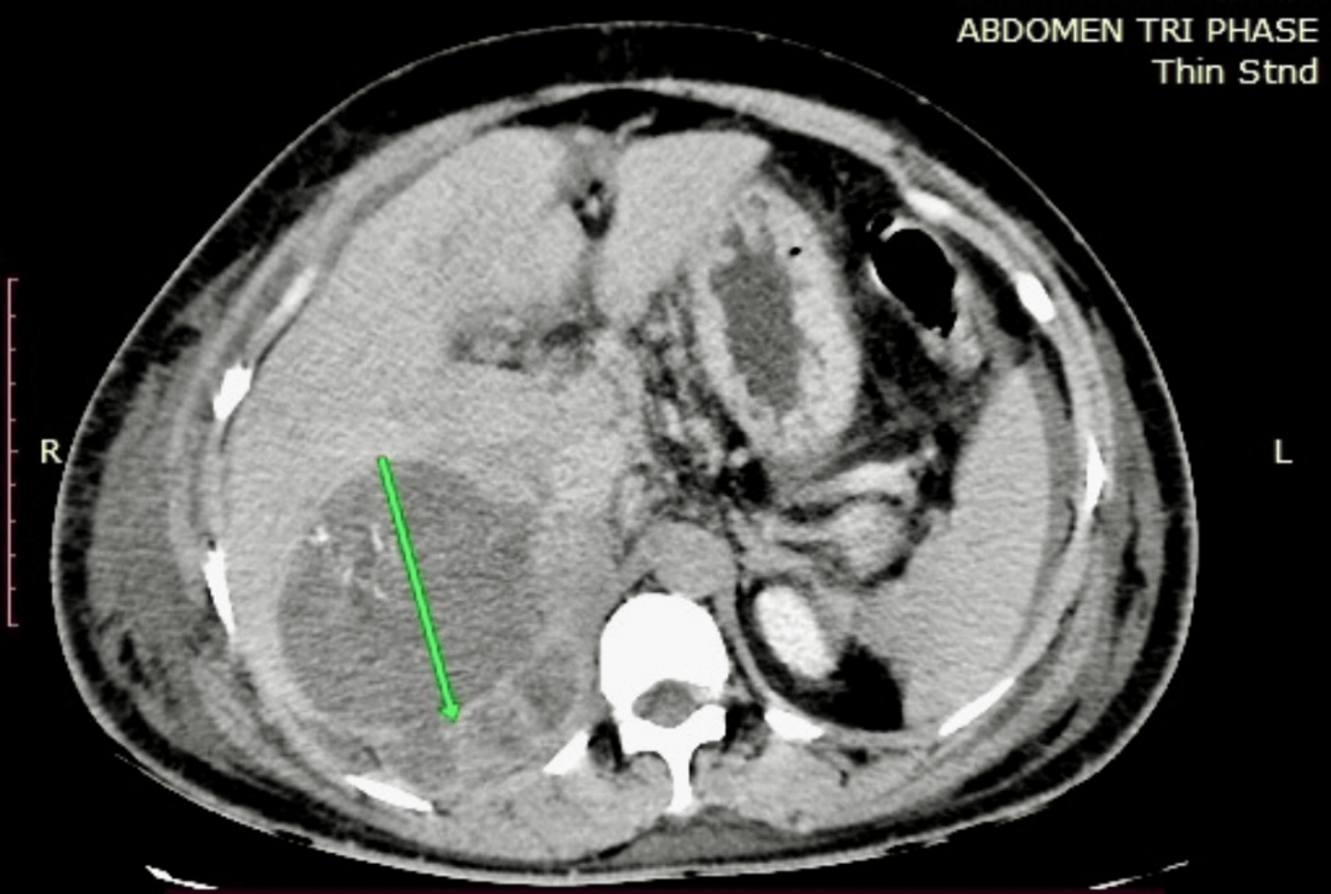
Coronal section of contrast-enhanced computed tomography (CECT) showing daughter cysts (arrow) within a primary hydatid cyst.

**Figure 2:**
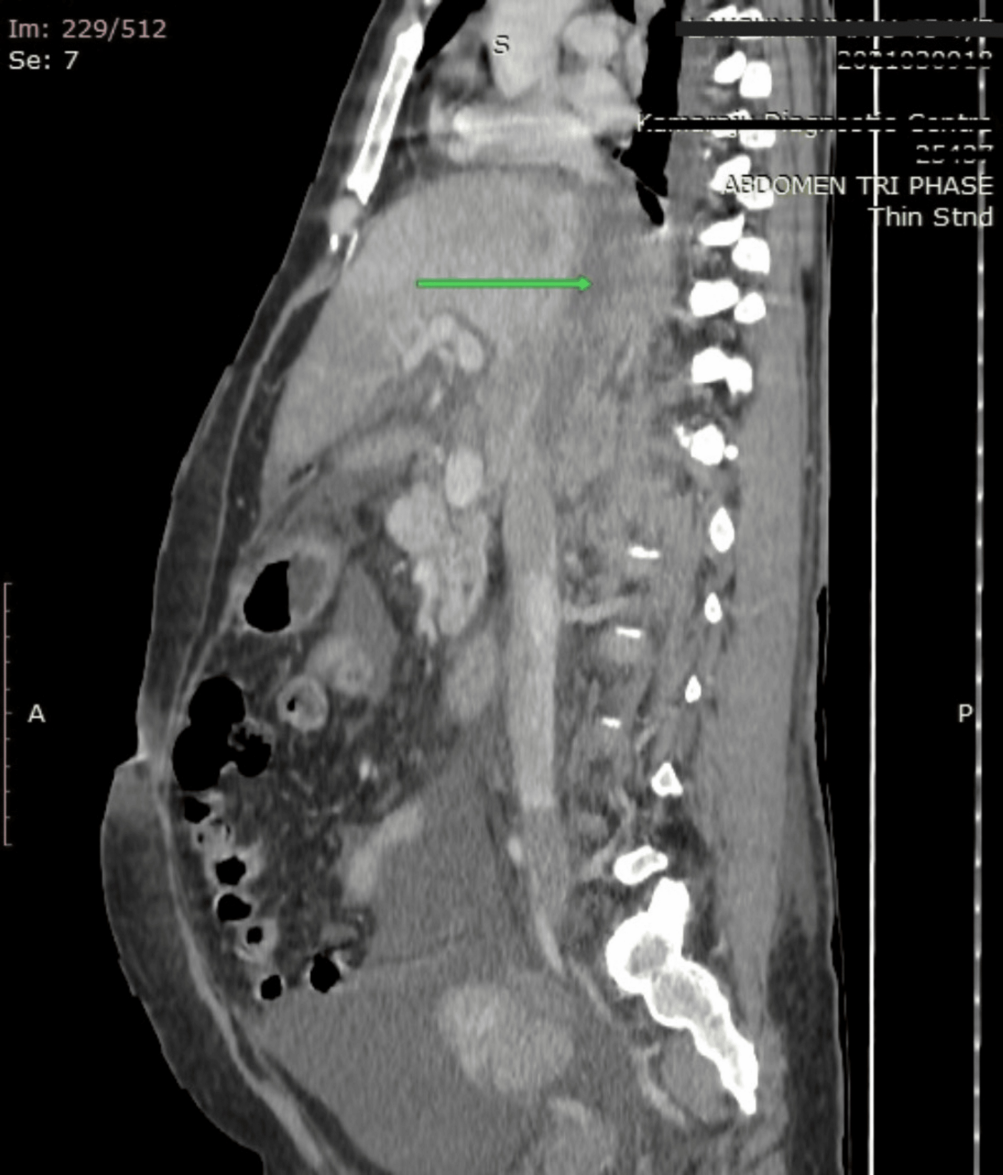
CECT abdomen showing thrombus (arrow) in retrohepatic IVC extending up to the right atrium.

On arrival, a 46-year-old woman with no known co-morbidities complained of generalized and non-specific pain in the abdomen for the last fortnight accompanied by abdominal tumescent over the past 10 days. For three days, she has also been experiencing emesis and vomiting non-bilious contents about three to four times per day. Physical findings revealed bilateral pitting edema on the feet and dilated veins on the posterior abdominal wall as observed during the abdominal inspection that revealed distension. On palpating, there was tenderness in the right hypochondrium, while hepatomegaly was noted via palpation at the right costal margin, which was 5 cm away from it; examination through percussion showed shifting dullness, and milking out of dilated veins indicated blood flow diverted away from the umbilicus; auscultation confirmed the presence of bowel sounds, whereas other systemic examinations were unremarkable: vitals including BP (110/70 mmHg), pulse rate (72 bpm), and jugular venous pressure (JVP) being normal. Her laboratory results (Table 1) indicated anemia (Hb: 10.3 g/dL), increased liver enzymes (AST: 311 U/L, ALT: 254 U/L), and hypoalbuminemia (Alb: 2.5 g/dL). USG abdomen showed heterogeneous lesions in the right lobe of the liver with calcifications, flow not visualized in hepatic veins, and IVC. Contrast-enhanced computed tomography (CECT) abdomen showed a cystic lesion likely a hydatid cyst with daughter cysts (Figure 1) and calcifications, thrombus of retrohepatic IVC (Figure 2), right and left hepatic veins, partial occluding thrombus in right common and external iliac veins with moderate ascites and mild pleural effusion bilaterally. 2D echo showed a thin layer of pericardial effusion with normal LV function.

**Table 1:** Laboratory values.

Investigation	Patient’s value	Reference value
Hemoglobin (Hb) (g/dL)	10.3	Male 13-17, Female 12-15
Total RBC Count (million/cu.mm)	4.1	Male 4.5-5.5, Female 3.8-4.8
Packed Cell Volume (PCV) (%)	34.6	Male 40-50, Female 36-46
Mean Corpuscular Volume (MCV) (fL)	79.7	83-101
Mean Corpuscular Hemoglobin (MCH) (pg)	23.8	27-32
Mean Corpuscular Hemoglobin Concentration(MCHC) (g/dL)	29.8	31.5-34.5
Red Cell Distribution Width (%)	16.2	11.6-14
Platelet Count (lakhs/cu.mm)	2.93	1.5-4.5
Total Leukocyte Count(TLC) (cells/cu.mm)	13000	4000-10000
Neutrophils(%)	70	40-80
Lymphocytes (%)	23.6	20-40
Monocytes (%)	5.1	2-10
Eosinophils (%)	0.5	1-6
Basophils (%)	0.7	<1-2
Absolute Neutrophil Count (ANC) (cells/cu.mm)	9080	2000-7000
Erythrocyte sedimentation rate (ESR )(mm/hr)	55	Male 17-60 years: <12, Male>60 years: 14-30, Female 17-60 years: <19 Female>60 years: 20-35)
Serum Sodium (meq/L)	133	137-145
Serum Potassium (meq/L)	4.7	3.5-5
Serum Chloride (meq/L)	99	96-106
Serum Bi-carbonate (meq/L)	21.1	22-26
Urea (mg/dL)	49	17-43
Creatinine (mg/dL)	1.3	0.7-1.4
Uric Acid (mg/dL)	8.8	2.5-7
Total Bilirubin (mg/dL)	0.63	0.2-1.3
Direct Bilirubin (mg/dL)	0.42	0.1-0.4
Total Protein (g/dL)	5.6	6-8
Aspartate Transaminase (AST) (IU/L)	311	14-36
Alanine Transaminaase (ALT) (IU/L)	254	5-50
Alkaline Phospatase (ALKP) (IU/L)	109	3.5-5
Albumin (g/dL)	2.5	3.5-5
Glyco Hb (HBA1C)	6.3	<5.7%
Random Blood Sugar (RBS) (mg/dL)	102	70-100
Urine routine	Normal	-
Ascitic fluid analysis	Color - red, Appearance - turbid, Total counts - 126, Neutrophils - 40, Lymphocytes - 60, Glucose - 121, Protein - 2 g, Albumin - 1 g, SAAG - 1.8, Others - plenty of RBC in background, Cytology - negative for malignant cells	-
Ascitic fluid culture	No growth	-
Blood culture and sensitivity	No growth	
Blood grouping and typing	O positive	-
Calcium (mg/dL)	8	9-11
Phosphorous (mg/dL)	4.6	3.4-5
Prothrombin Time (PT) (sec)	17.1	10-14
International Normalized Ratio (INR)	1.51	0.9-1.1
Activated Partial Thromboplastin Time (APTT) (sec)	25.7	21-34
Echinococcus IgG antibody	Positive	Negative

Hydatid serology for IgG antibody was positive, and ascitic fluid analysis showed a serum ascites albumin gradient (SAAG) ratio of 1.8 with negative culture and malignant cells. This patient was then treated with albendazole, therapeutic paracentesis, and anticoagulation therapy with an injection of heparin 5,000 IU every six hours. However, surgery that involved cyst resection was disallowed by the patient. The patient was discharged against medical advice. During the follow-up, it was revealed that the patient experienced an unexpected cardiopulmonary arrest three weeks after discharge and subsequently expired.

## Discussion

BCS is a comparatively rare and perilous condition that stems from occlusion of the hepatic venous outflow tract, leading to increased portal vein pressure, liver congestion, and potential acute liver failure. Nonetheless, in developing countries, the cause might be more often due to other things such as parasitic infections or hematological disorders [6]. The case report is significant because it describes an unusual cause of secondary BCS: compression of the hepatic veins and inferior vena cava (IVC) by a hydatid cyst. Echinococcus tapeworm-induced hydatid disease is endemic in areas where sheep-rearing is common, especially in parts of the Middle East, Africa, South America, and Central Asia [7]. Normally asymptomatic until they reach considerable size or complicate into rupture or secondary infection or as in this case compressing adjacent structures; hepatitic hydatids cysts often remain silent until that time comes. However, it is extremely rare for BCS to arise from a hydatid cyst. Hepatic hydatid cysts account for about 12% of cases associated with parasitic infections, which makes it an important but not common cause of this syndrome [8]. In this context, the pathophysiology of BCS refers to mechanical compression by an enlarging hydatid cyst on the hepatic veins and IVC leading to venous outflow obstruction from the liver. This results in an increased sinusoidal pressure leading to hepatic congestion with hepatomegaly leading to ascites and impaired liver function. In addition, the patient had other symptoms such as nausea and vomiting that were due to the presence of hydatid cysts besides having the typical signs of BCS, including abdominal distension and pain [9]. Various methods such as USG, CT scan, or MRI are usually employed to confirm hepatic hydatid cyst diagnosis showing characteristic cystic lesions within the liver. Additionally, serological tests using enzyme immunoassay (EIA) for Echinococcus antibodies help confirm the diagnosis. The imaging, in this case, showed hepatomegaly with a heterogeneously attenuating lesion in the right lobe of the liver, calcifications within it, and a complete lack of flow signal in both hepatic veins and IVC. These findings along with hydatid serology positivity confirmed secondary BCS due to hydatid disease [10]. Management of hydatid cysts includes anti-parasitic therapy along with surgical interventions if required such as surgical resection of the cyst or percutaneous aspiration of the cyst and instilling scolicidal agents for managing complications including BCS, as illustrated in this case. For instance, benzimidazole carbamate, albendazole, is usually prescribed for most patients suffering from hydatidosis. It works by impairing "glucose uptake and glycogen storage" belonging to humans or parasites. However, definitive treatment of hepatic hydatid cysts often requires surgical resection, especially when they become large or lead to severe complications, such as BCS; however, despite the recommendation for surgical resection, the patient declined this procedure indicating that clinicians face challenges when patients refuse or cannot undergo necessary interventions [11]. Albendazole and anticoagulation were therefore used as conservative management measures to reduce the likelihood of further thrombotic events. Moreover, we performed paracentesis for therapeutic purposes to relieve the symptoms associated with ascites. However, despite all these precautions, three weeks after discharge, the patient had a sudden cardiac death [12]. According to the research, anaphylactic shock can happen when a hydatid cyst bursts either spontaneously or due to trauma, causing a sudden release of its fluid that contains antigenic material. This anaphylaxis could be fatal if it is not recognized and managed soon enough probably explaining why this patient suddenly died. Furthermore, surgical treatment should always be considered in any patient presenting with complicated cysts, multiple daughter cysts, or superficial hydatid cysts, which are at high risk of rupture because their rupture could cause an anaphylactic reaction, which is fatal.

## Conclusions

This case underscores the importance of considering hydatid cysts as a differential diagnosis when evaluating patients with BCS. Hepatic hydatid cysts, caused by the parasitic infection of Echinococcus granulosus, are relatively rare but can present in a manner that mimics or contributes to BCS. The particularity of this case - where a hepatic hydatid cyst led to the development of BCS - highlights a crucial point: the need for clinicians to maintain a high index of suspicion for such parasitic infections in patients who present with unexplained hepatic abnormalities. Early identification of hydatid cysts is vital as it can significantly influence treatment decisions and potentially improve patient outcomes. The unfortunate outcome of this patient, despite therapeutic interventions, serves as a stark reminder of the challenges associated with managing rare and complex hepatic conditions. This case illustrates that timely and accurate diagnosis, alongside appropriate surgical and medical management, is essential to mitigate the risks of severe complications such as BCS. The complexities involved in treating such cases emphasize the necessity for a multidisciplinary approach and rigorous diagnostic evaluation to prevent similar adverse outcomes in the future.
